# Facilitation through altered resource availability in a mixed‐species rodent malaria infection

**DOI:** 10.1111/ele.12639

**Published:** 2016-06-30

**Authors:** Ricardo S. Ramiro, Laura C. Pollitt, Nicole Mideo, Sarah E. Reece

**Affiliations:** ^1^ Institutes of Evolutionary Biology, and Immunology and Infection ResearchUniversity of EdinburghEdinburghEH9 3JFLUK; ^2^ Centre for Immunity, Infection & EvolutionSchool of Biological SciencesAshworth LaboratoriesUniversity of EdinburghEdinburghEH9 3JFLUK; ^3^ Department of Ecology and Evolutionary BiologyUniversity of TorontoTorontoCanada

**Keywords:** Co‐infection, facilitation, genetically diverse infection, Malaria, *Plasmodium chabaudi*, *Plasmodium yoelii*, red blood cell, reticulocyte, species interactions, virulence

## Abstract

A major challenge in disease ecology is to understand how co‐infecting parasite species interact. We manipulate *in vivo* resources and immunity to explain interactions between two rodent malaria parasites, *Plasmodium chabaudi* and *P. yoelii*. These species have analogous resource‐use strategies to the human parasites *Plasmodium falciparum* and *P. vivax*:* P. chabaudi* and *P. falciparum* infect red blood cells (RBC) of all ages (RBC generalist); *P. yoelii* and *P. vivax* preferentially infect young RBCs (RBC specialist). We find that: (1) recent infection with the RBC generalist facilitates the RBC specialist (*P. yoelii* density is enhanced ~10 fold). This occurs because the RBC generalist increases availability of the RBC specialist's preferred resource; (2) co‐infections with the RBC generalist and RBC specialist are highly virulent; (3) and the presence of an RBC generalist in a host population can increase the prevalence of an RBC specialist. Thus, we show that resources shape how parasite species interact and have epidemiological consequences.

## Introduction

Mixed‐species infections are common and interactions between co‐infecting species can either promote or inhibit other parasite species in the same host (Graham 2008; Griffiths *et al*. 2014). These interactions affect disease severity, parasite fitness and the prevalence and distribution of parasite species in a host population (Ferrari *et al*. 2009; Knowles *et al*. 2013; Pedersen & Antonovics 2013; Viney & Graham 2013). However, the within‐host mechanisms that underpin between‐species interactions and drive their epidemiological consequences are poorly understood. Co‐infecting parasite species can interact with one another directly or, more commonly, through changing the within‐host environment (Mideo 2009). For example, the nematode *Nippostrongylus brasiliensis* alters resources available for *Plasmodium chabaudi* (rodent malaria) during co‐infection of mice (Griffiths *et al*. 2015). Interactions also occur via the host immune response if one species interferes with, or enhances, attack on a co‐infecting species (Wolday *et al*. 1999; Reese *et al*. 2014). Determining how interactions between co‐infecting parasite species shape infection dynamics, virulence and transmission are major questions in disease ecology.

Malaria infections in humans, rodents and birds commonly contain multiple co‐infecting parasite species (Killick‐Kendrick & Peters 1978; Valkiunas *et al*. 2006; Juliano *et al*. 2010). For example, between 5 and 65% of human malaria infections contain both *Plasmodium falciparum* and *Plasmodium vivax* parasites (Looareesuwan *et al*. 1987; Mayxay *et al*. 2004; Douglas *et al*. 2011; Ginouves *et al*. 2015). Interactions between conspecific genotypes result in competitive suppression in the rodent malaria *P. chabaudi,* and this has significant implications for the evolution of both virulence and drug resistance (Bell *et al*. 2006; Pollitt *et al*. 2011, 2014; Read *et al*. 2011). Correlational data suggest that competitive suppression also occurs between conspecific genotypes of *P. falciparum* (Daubersies *et al*. 1996; Mercereau‐Puijalon 1996; Smith *et al*. 1999; Färnert 2008). However, whereas competition has been demonstrated between conspecifics, whether this also occurs between heterospecifics within mixed‐species infections is poorly understood (but see (Bruce *et al*. 2000)).

Dynamics of malaria infections are shaped through complex combinations of top‐down (immunity) and bottom‐up (resources) processes (Haydon *et al*. 2003; Mideo *et al*. 2008; Metcalf *et al*. 2011). Some species of malaria parasites preferentially infect particular age classes of red blood cells (RBC), so different species may occupy slightly separate resource niches in the host. As well as reducing resource competition, it has been suggested that this niche differentiation could lead to facilitation. Mcqueen & Mckenzie (2006) modelled parasite and RBC dynamics in co‐infections of the two most common human malaria parasite species and predicted that *P. falciparum* facilitates replication of *P. vivax*. This occurs because *P. falciparum* (a generalist that infects RBCs of all ages) induces anaemia which causes the host to produce new RBCs and shifts the age structure of host RBCs towards younger cells, which are the preferred resource of *P. vivax* (an RBC specialist; Mcqueen & Mckenzie 2006). If an RBC‐specialist species benefits from the presence of an RBC generalist, the consequences of infection by the specialist, for individual hosts and at the population level, will depend on the presence of the generalist. Thus, in areas where *P. falciparum* prevalence is declining (e.g. through control programmes) this may cause unintended changes to *P. vivax* prevalence.

Here, we test the predictions of Mcqueen & Mckenzie (2006) by experimentally perturbing resource availability and the immune environment (apparent competition) of mixed‐species malaria infections. Specifically, we test whether one species of rodent malaria parasite (*P. chabaudi*) can alter RBC resource availability sufficiently to facilitate the replication of another species (*P. yoelii*). *P. chabaudi* is an RBC generalist because it infects RBCs of all ages, whereas *P. yoelii* is a RBC specialist that preferentially infects young RBCs. These species are ideal model systems for investigating the within‐host mechanisms mediating interactions between parasites because the ecology of individual species is well understood, the performance of each species can be tracked and RBC resources and immunity can be separately perturbed. We show that the RBC‐specialist parasite (*P. yoelii*) reaches higher densities in hosts that have an existing infection with the RBC generalist (*P. chabaudi*). This facilitation occurs due to an increase in the supply of the specialist's preferred age of RBC, with heterologous immunity (raised against the heterospecific *P. chabaudi*) having little impact on *P. yoelii* densities. Furthermore, we show that mixed‐species infections increase the risk of host mortality. Motivated by our findings, we develop a heuristic model of the fitness of an RBC‐specialist parasite in different within‐host environments to determine the conditions under which fitness will be higher if an RBC generalist is also circulating in the host population. We find that the facilitation we observe only benefits *P. yoelii* when the prevalence of *P. chabaudi*, at the host population level, is below a particular threshold. Overall, our results reveal how multiple parasite species, with differing resource preferences, interact through bottom‐up changes to the within‐host environment, impacting upon parasite replication, host health and epidemiology.

## Materials and methods

### Parasites

We used the parasite genotypes: *P. chabaudi chabaudi* AS (AS12476) and *P. yoelii yoelii* 17X Mill Hill (35GA), from the European Malaria Reagent Repository, University of Edinburgh. Both species were isolated from thicket rats in Central African Republic during the 1960s and were often (12/22 cases) found to co‐infect the same host. Whereas *P. chabaudi chabaudi* can infect RBCs of all age classes (RBC generalist), *P. yoelii yoelii* shows strong preference for the youngest RBCs (reticulocytes; RBC specialist). Both parasite species have been widely used to address questions ranging from the molecular mechanisms of RBC invasion to the competitive dynamics between conspecific strains.

### Infections

Hosts were 8–10‐weeks‐old male MF1 mice (Harlan‐Olac, Bicester, UK), maintained on *ad libitum* food (RM3(P), DBM Scotland Ltd, Grangemouth, UK) and water (supplemented with 0.05% PABA to enhance parasite growth), with a 12 : 12 h light:dark cycle, at 21 °C. Mice were randomly allocated to cages containing 2–4 animals and randomly assigned to treatment groups. Infections were initiated by intraperitoneal (IP) injection of 10^5^ parasitised RBCs in 100 μL carrier (following Bell *et al*. 2006) and mixed‐species infections with 2 × 10^5^ infected RBCs in 200 μL carrier, consisting of 10^5^ infected RBCs of each parasite species (note that increasing the initial dose by a factor of > 10 is required to significantly affect parasite dynamics and virulence; Timms *et al*. 2001). Our design keeps the dose of the focal species (*P. yoelii*) constant while altering the within‐host environment, a standard practice when studying *Plasmodium* co‐infections (Råberg *et al*. 2006; Reece *et al*. 2008; Pollitt *et al*. 2011). We chose *P. yoelii* as the focal species because our work is motivated by Mcqueen & Mckenzie (2006), in which the human parasite *P.falciparum* (RBC generalist) is predicted to facilitate *P. vivax* (RBC specialist). Protocols passed ethical review and are approved by the UK Home Office (Project License 60/4121). All procedures were carried out in accordance with the UK Animals (Scientific Procedures) Act 1986.

### Experimental design

**Figure 1:**
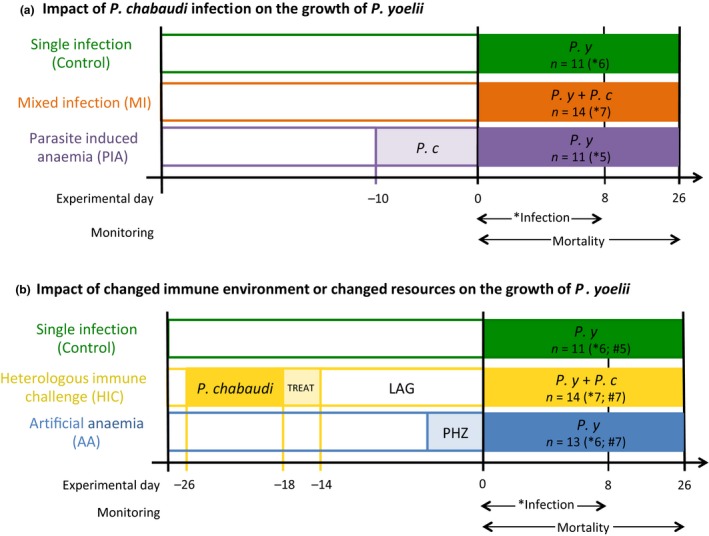
Experimental design and treatment groups. Schematic of the experimental timeline for testing the impact of *P*. chabaudi infection on the growth of *P*. yoelii (A) or the impact of changed immune environment or changed resources on the growth of *P*. yoelii (B). Treatment groups are illustrated according to their analysis for clarity, though all infections occurred in parallel and the control group was the same for all analyses. Sample sizes are shown for each treatment in the following format; *n* = number of mice monitored for mortality and number of infections used in parasite density analyses (* tracked by PCR; # tracked by microscopy).

We designed our experiments to test the impact of prior or concurrent infection with an RBC‐generalist species on the replication of an RBC specialist and determine the contributions of the immune and resource environments to the RBC‐specialist's performance. Mice were allocated to one of the five following treatment groups: (1) Single infection (control) mice received only *P. yoelii;* (2) Mixed infection (MI) mice simultaneously received *P. yoelii* and *P. chabaudi;* (3) Parasite‐Induced Anaemia (PIA) mice were infected with *P. chabaudi* 10 days before they received *P. yoelii*; (4) Heterologous Immune Challenge (HIC) mice were infected with *P. chabaudi*, cured after 8 days by IP injection of 12 mg kg^−1^ pyrimethamine (in 50 μL DMSO; Sigma, Dorcet UK) for two consecutive days, and then given pyrimethamine‐treated water (7 mg mL^−1^) for 2 more days. After 14 days, when RBC age structure and density had returned to pre‐infection levels, antibodies against *P. chabaudi* had developed and all *P. chabaudi* parasites were cleared (confirmed by qPCR), these mice were infected with *P. yoelii*; (5) Artificial Anaemia (AA) mice were IP injected with 60 mg/kg^−1^ phenylhydrazine (PHZ; dissolved in PBS; Sigma, UK) 3 days before infection with *P. yoelii*. PHZ induces a large increase in reticulocytes (*P. yoelii*'s preferred resource) within days (Savill *et al*. 2009). All infections with *P. yoelii* were initiated at the same time from the same parasite inoculum and 5–7 mice were infected for each treatment. To control for any possible effects of pyrimethamine in the HIC treatment group, mice in all groups were treated with pyrimethamine, in the same manner, prior to *P. yoelii* infection. To control for *P. chabaudi* or PHZ injections, we injected mice in the groups not receiving these treatments with the carrier solution plus uninfected RBCs or PBS, respectively. All treatment groups, sample sizes and the experimental timeline are displayed in Fig. 1.

### Sampling

We used species‐specific qPCR to separately count the number of *P. yoelii* and *P. chabaudi* genomes per μL of blood. *P. yoelii* cumulative density was calculated from samples taken from each mouse on alternate days between day 0 and day 8 post‐infection. This period was chosen because we are interested in how the starting conditions of the within‐host environment impact the replication of our focal (RBC specialist) parasite species (Fig. S5 for *P. yoelii* temporal dynamics). Because *P. yoelii* replication is non‐synchronous, PCR counts could overestimate the number of infected RBCs if schizonts (parasites in the final stage of their cell cycle, after DNA replication has occurred) are present or if RBCs are parasitised by multiple parasites. To check for this, we also calculated the density of *P. yoelii‐*infected RBCs from slides for single‐species infections (treatments: control, HIC and AA). Overall, *P. yoelii* densities were slightly lower when calculated from slides, but the relative difference between treatment groups was consistent with counts by PCR (Treatment × measurement method; F_2,32_ = 0.8, *P* = 0.46; Table S2). Thus, for analyses of single‐species infections we include *P. yoelii* densities estimated through both PCR and microscopy (while controlling for method). On each sampling day, mice were weighed, blood samples were taken from the tail for qPCR of parasite density (5 μL) and to quantify RBCs by flow cytometry (2 μL; Beckman Coulter), and thin blood smears were taken to determine the proportion of RBCs that were reticulocytes. To estimate heterologous and homologous immunity on the day of *P. yoelii* infection, we assayed IgG2a antibodies (10 μL of blood), which are known to induce strong protection against malaria in mice (Cavinato *et al*. 2001). We measured homologous immunity against a particular region of MSP1 which is specific for *P. chabaudi* AS (O'Donnell *et al*. 2001; Burns *et al*. 2004; Fairlie‐Clarke *et al*. 2010) and heterologous immunity against crude antigen homogenate from *P. yoelii‐*parasitised RBCs. This verified that prior infection with *P. chabaudi* generated an acquired response and gave a measure of antibodies that could also act against *P. yoelii* (i.e. species‐transcending antibodies generated by *P. chabaudi*). Further details for the qPCR and immunological assays are given in SI.

### Data analyses

All analyses were performed using R v3.0.2 (R core team (2013) The R foundation for statistical computing; http://www.R-project.org). General linear models, combined with post‐hoc Tukey contrasts (package multcomp), were used to test for treatment effects on the following response variables: cumulative *P. yoelii* density (log10 transformed), day 0 RBC density (log10 transformed), day 0 reticulocyte density (log10 transformed), proportion reticulocytes (logit transformed) and antibody titres (log10 transformed). To determine the relative contributions of within‐host variables to cumulative *P. yoelii* density (log10 transformed), we used a general linear model with all the explanatory variables described above. We used a generalised linear model (with binomial error structure) to test for the effects of treatment, minimum RBC density (log10 transformed), mean proportion of reticulocytes and minimum weight on host mortality. We followed model simplification, sequentially dropping the least significant term until the minimum adequate model was reached. Full details of all statistical models are provided in Tables S1–6.

### Modelling

To predict how the prevalence of *P. chabaudi* infections in a host population affects *P. yoelii* fitness, we developed a simple mathematical model. We divide the host population into three classes, corresponding to three of our experimental treatment groups: naïve hosts who have never been infected by *P. chabaudi* (e.g. control; C), hosts who are currently infected by *P. chabaudi* and whose within‐host environment has yet to be altered (e.g. MI) and hosts who are recovering from a *P. chabaudi* infection and have an altered within‐host environment (e.g. PIA). Assuming *P. yoelii* is equally likely to be transmitted to each host type, then the average fitness of the parasite will be the sum of the fitness achieved in each host type, *w*_*i*_, weighted by the frequency of that host type, *p*_*i*_. In other words,

(1) $$\overset{¯}{w}=p_{C}w_{C}+p_{\mathrm{MI}}w_{\mathrm{MI}}+p_{\mathrm{PIA}}w_{\mathrm{PIA}}$$

where the overbar denotes an average. We use ϕ to denote the proportion of the population that has ever been exposed to *P. chabaudi* (akin to ‘seroprevalence’; hosts in MI and PIA classes), and ψ to denote the prevalence of active *P. chabaudi* infections (MI only, which assumes that *P. chabaudi* densities are negligible in PIA hosts; note that we could recast the model with prevalence including PIA and MI hosts with no qualitative change in inferences). The frequencies of the host types are therefore,

$$p_{C}=\left(1-\phi \right),$$

$$p_{\mathrm{MI}}=\psi ,$$

$$p_{\mathrm{PIA}}=\phi -\psi .$$

We define the host type‐specific fitnesses (*w*_*i*_) according to the costs and benefits we observe in our experimental work and use this model to determine the conditions under which an RBC specialist (*P. yoelii*) is predicted to benefit from the presence of an RBC generalist (*P. chabaudi*) circulating in the host population.

## Results and discussion

We use data from different treatment groups to address two questions. First, does prior infection with an RBC generalist facilitate growth of an RBC specialist and does timing matter (Fig. 1a)? Second, how do changes in the immune environment and RBC resource availability independently affect replication of the RBC specialist (Fig. 1b)?

### Infection with an RBC generalist facilitates an RBC specialist

**Figure 2:**
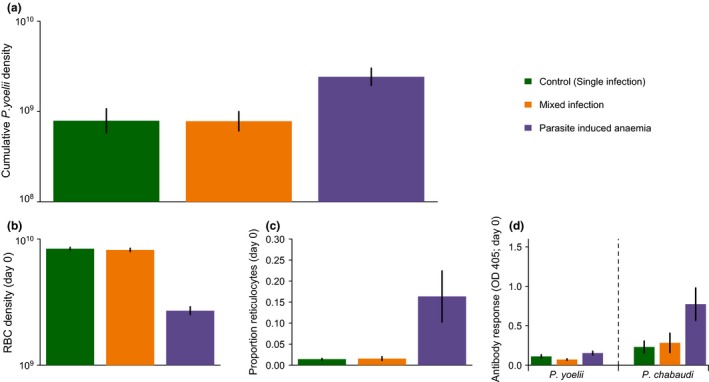
Infection with a red blood cell generalist facilitates replication of a specialist. Panel (a) shows *P. yoelli* density when infections are initiated alone in naïve hosts (control), at the same time as a *P. chabaudi* infection (mixed infection) or 10 days after a *P. chabaudi* infection (parasite‐induced anaemia). Panels (b–d) illustrate how the within‐host environment on the day of *P. yoelli* infection differs between treatment groups. Error bars show the standard error of the mean.

When *P. yoelii* parasites were inoculated into a within‐host environment that had already been altered by *P. chabaudi* infection, they reached significantly higher densities than in either control infections or simultaneous mixed infections (PIA vs. control; z = 2.9, *P* = 0.01; PIA vs. MI; z = 2.98, *P* = 0.01; Fig. 2a; Table S2). *P. yoelii* parasites in simultaneous mixed infections had similar density to those in control infections (control vs. MI; z = 0.03, *P* = 0.9994; Fig. 2a; Table S2). Therefore, a recent infection with an RBC generalist (*P. chabaudi*) facilitates a reticulocyte specialist (*P. yoelii*). However, timing matters because significant facilitation only occurred when the RBC generalist was already established in the host.

The difference in performance of *P. yoelii* across treatments can be explained by the within‐host environment at infection. Pre‐infection with *P. chabaudi* (but not simultaneous infection) significantly reduced the total RBC density of hosts (PIA vs. control; z = 22.8, *P* < 0.0001; Table S1; Fig. 2b), but significantly increased the density and proportion of reticulocytes (PIA vs. control for reticulocyte density: z = 3.7, *P* = 0.002; and proportion of reticulocytes: z = 7.6, *P* < 0.001; Table S1; Fig. 2c) compared to control mice, on the day of infection with *P. yoelii*. Pre‐infection with *P. chabaudi* also led to an increase in the concentration of antibodies to *P. chabaudi* (PIA vs. control; z = 3.1, *P* = 0.02; Table S1; Fig. 2d), but antibodies to *P. yoelii* were unchanged (treatment, F = 2.15, *P* = 0.09; Table S1; Fig. 2d). In contrast, on the day of infection, mice simultaneously receiving *P. yoelii* and *P. chabaudi* (MI treatment group) did not differ significantly from control mice in either resources or immunity (RBC density: z = 0.51, *P* = 0.99; reticulocyte density: z = 0.21, *P* = 0.99; proportion of reticulocytes: z = 0.12, *P* = 0.99; *P. yoelii* antibodies: treatment, F = 2.15, *P* = 0.09; *P. chabaudi* antibodies: z = 0.44, *P* = 0.99; Table S1; Fig. 2). These results suggest that the facilitation observed in the PIA group is mediated by host anaemia resulting in increased production of reticulocytes (Mcqueen & Mckenzie 2006).

### Resource availability is a key determinant of the RBC‐specialist's performance

The most notable change to the within‐host environment in the infections where *P. yoelii* was facilitated is the substantial increase in the density and proportion of RBCs that are reticulocytes (Figs 2 and S3). However, it is also possible that pre‐infection with *P. chabaudi* benefits *P. yoelii* parasites by altering the host's immune environment in a manner not reflected in the antibody assays, or that the few remaining *P. chabaudi* parasites (Fig. S1) interacted directly with *P. yoelii* parasites. The artificial anaemia and heterologous immunity treatment groups allow us to partition the relative contributions of RBC resources and heterologous immunity to the facilitation of *P. yoelii* (below and Fig. 1b).

**Figure 3:**
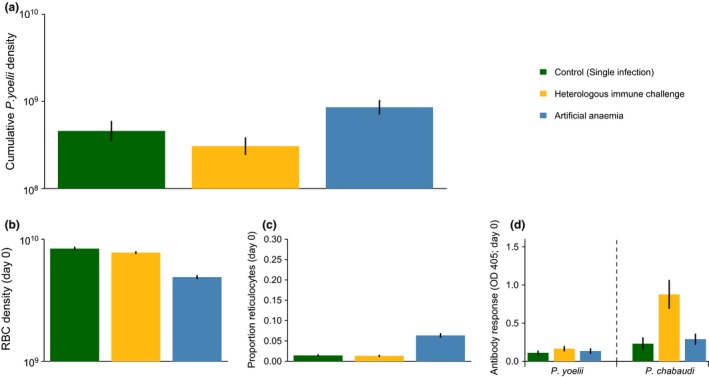
Resource availability is a key determinant of *P. yoelii* success. Panel (a) shows *P. yoelli* density when infections are initiated alone in naïve hosts (control), in a host with a previously cleared *P. chabaudi* infection (heterologous immune challenge) or in a host treated with PHZ (artificial anaemia). Panels (b–d) illustrate how the within‐host environment on the day of *P. yoelli* infection differs between treatment groups. Error bars show the standard error of the mean.

In the artificial anaemia treatment group, mice were treated with PHZ prior to *P. yoelii* infection. PHZ causes anaemia, resulting in lower total RBC density (control vs. AA; z = 14.2, *P* < 0.001; Table S1; Fig. 3b), and stimulates the production of reticulocytes (proportion of reticulocytes: z = 6.7, *P* < 0.001; reticulocyte density: control vs. AA; z = 4.6, *P* < 0.001; Table S1; Figs 3c and S3). In comparison to the parasite‐induced anaemia group, the overall reduction in RBC density in the artificial anaemia group was lower (PIA vs. AA; z = 12.3, *P* < 0.001), and there was a borderline increase in the proportion of reticulocytes (mean reticulocyte proportion: PIA = 0.16 (±0.06), AA = 0.06 (±0.004); z = 2.5, *P* = 0.08), but the resulting reticulocyte density was nearly identical (mean log_10_ reticulocyte density: PIA = 8.51 (±0.16), AA = 8.48 (±0.02); z = 0.3, *P* = 0.99). As intended, PHZ treatment did not alter the concentration of antibodies to *P. yoelii* or *P. chabaudi* compared to control mice (*P. yoelii* antibodies: treatment, F = 2.15, *P* = 0.09; *P. chabaudi* antibodies: AA vs. control, z = 2.3, *P* = 0.14; Fig. 3d). As predicted under the hypothesis that elevating reticulocytes facilitates an RBC specialist, *P. yoelii* density was nearly two times higher in the artificial anaemia treatment than in control mice (z = 2.6, *P* = 0.025).

In the heterologous immunity treatment group, mice were given an infection with *P. chabaudi* 26 days prior to inoculation with *P. yoelii*. *P. chabaudi* parasites were allowed to establish for 8 days before receiving drug treatment to clear all parasites. The density and age structure of RBCs had returned to normal by the time of *P. yoelii* infection (control vs. HIC on day 0: RBC density, z = 2.03, *P* = 0.25; proportion of reticulocytes: z = 0.25, *P* = 0.99; reticulocyte density, z = 0.59, *P* = 0.98). As intended, pre‐infection with *P. chabaudi* resulted in significantly higher anti‐*P. chabaudi* antibodies than in control mice (z = 3.9, *P* < 0.001), as measured through the IgG2a antibodies targeting *P. chabaudi* MSP1. In contrast, antibodies against *P. yoelii* crude antigen remained unchanged (treatment, F = 2.15, *P* = 0.09), suggesting that *P. chabaudi* infection did not generate a species‐transcending IgG2a response. As predicted, *P. yoelii* density in HIC mice was not significantly different from controls (z = 1.4, *P* = 0.34).

The results above strongly support the role of resource availability in facilitating *P. yoelii* and suggest that heterologous immunity plays a minor (if any) role. Although the reticulocyte density on the day of infection was nearly identical under the parasite‐induced anaemia and artificial anaemia treatments, the relative increase in parasite density was slightly higher in parasite‐induced anaemia mice. This could be due to the proportion of RBCs that were reticulocytes being higher, or to other differences in the within‐host environment (e.g. immunity). To further investigate whether any of these variables could cause the increase in *P. yoelii* density in anaemic mice, we analysed data from PCR counts (to ensure only *P. yoelii* was counted) for all treatments. We fitted a model with the following explanatory variables measured on day 0: reticulocyte density, proportion of RBC that were reticulocytes and levels of antibodies to *P. chabaudi* and *P. yoelii*. The proportion of reticulocytes was the only variable that showed a significant positive correlation with *P. yoelii* density (F_1,29_ = 6.4, *P* = 0.017; Fig. S2; Table S3), further suggesting that resources, not immunity, are the drivers of facilitation. Because reticulocyte density failed to explain significant variation in *P. yoelii* density (F_1,26_ = 0.05, *P* = 0.82), the relative abundance of reticulocytes rather than their absolute density may determine *P. yoelii* replication rate. All malaria parasites have limited time to find a suitable RBC to invade and the encounter rate with their preferred resource is likely to be crucial for infection success (Cowman & Crabb 2006).

### Co‐infections of an RBC generalist and RBC specialist have a high virulence

Hosts with a single infection of either our RBC‐generalist or our RBC‐specialist species usually control their infection through immunity and replacement of lost RBC (Spence *et al*. 2013; Pollitt *et al*. 2015). However, in the facilitated infections, the production of RBC fuelled the RBC specialist and heterologous immunity had no impact. We tested whether these processes resulted in mixed‐species infections being more virulent than single‐species infections. We compared mortality, weight loss and anaemia for the mice used in the previous analyses plus an additional 5–7 mice per treatment (see Fig. 1) for 26 days after infection with *P. yoelii*. Weight loss and anaemia are standard measures of virulence in this system (De Roode *et al*. 2005; Bell *et al*. 2006; Pollitt *et al*. 2013, 2014, 2015).

**Figure 4:**
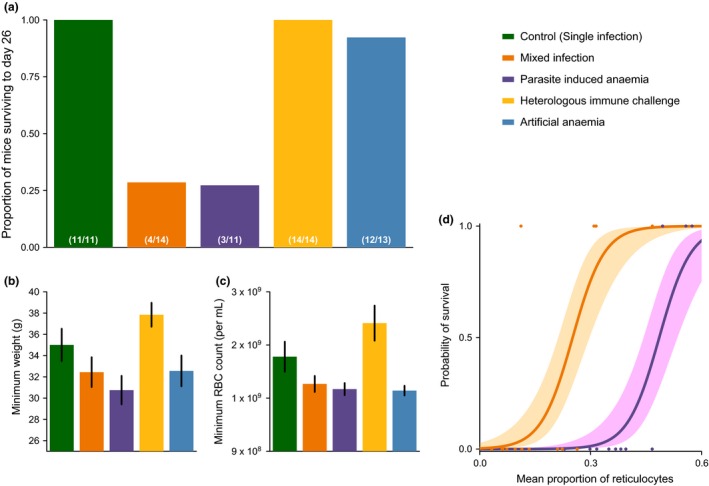
Co‐infections of a RBC generalist and RBC specialist are more virulent to the host. Panels (a, b and c) show the proportion surviving mice, minimum weight and minimum red blood cell count for the five treatment groups, respectively. Panel d shows the relationship between mean proportion of reticulocytes and survival for the two treatments where substantial mortality was experienced (mixed infections, orange, and parasite‐induced anaemia, purple).

Mortality differed dramatically among treatment groups (survival to day 26 ~ treatment: χ^2^_4,63_ = 23.11, *P* < 0.0001; Fig. 4a) with high mortality in the parasite‐induced anaemia and mixed infection treatments (71 and 80%, respectively), but few or no deaths in other treatment groups (control = 0%, HIC = 0%, AA 8%). Note that we did not investigate single infections with *P. chabaudi* because multiple experiments demonstrate that this species (and the particular strain we used) rarely leads to mouse mortality (Seixas & Ostler 2005; Bell *et al*. 2006; Mideo *et al*. 2008; Reece *et al*. 2008; Pollitt *et al*. 2011). Therefore, infection with two parasite species, either simultaneously or consecutively, substantially increased the risk of host death (single infection vs. MI or PIA: χ^2^_1,63_ = 21.01, *P* < 0.0001; Table S4; Fig. S4, for survival temporal dynamics). This is in keeping with reports that mixed‐species infections of *P. falciparum* and *P. vivax* more often lead to severe disease symptoms in human malaria cases (Genton *et al*. 2008). Weight loss and anaemia differed significantly across treatments (minimum RBC count: F_4,63_ = 6.2, *P* < 0.0001; minimum weight: F_4,63_ = 4.15, *P* < 0.01) and this was driven by mice in the HIC becoming less anaemic and losing less weight than mice in PIA and AA treatment groups (Fig. 4b,c; all other pairwise comparisons between treatments were non‐significant (*P* > 0.05) for both minimum RBC density and minimum weight; Table S5).

The high mortality in the parasite‐induced anaemia and mixed infection treatment groups cannot be explained by weight loss or anaemia (minimum weight: χ^2^_1,20_ = 0.18, *P* = 0.67; minimum RBC density: χ^2^_1,21_ = 3.49, *P* = 0.062). Instead, we found a significant positive correlation between the mean proportion of RBC throughout infections which were reticulocytes and the probability of surviving (χ^2^_1,22_ = 15.2, *P* < 0.0001; Fig. 4d). We also found an effect of treatment (PIA vs. mixed: χ^2^_1,22_ = 8.38, *P* < 0.005; Table S6): mice with a simultaneous mixed infection had a higher probability of survival at a given reticulocyte proportion than mice in the parasite‐induced anaemia treatment (Fig. 4d). We conclude that hosts face a trade‐off: the production of new RBCs facilitates replication of *P. yoelii*, but new RBCs must be produced in large numbers to survive and recover from infection.

### The RBC specialist benefits when an RBC generalist is below a prevalence threshold

Motivated by our experimental results, we defined the fitness of RBC‐specialist (e.g. *P. yoelii*) parasites infecting the three host classes in our heuristic model. As a proxy for fitness, we used the ‘transmission potential’ of an infection, which is the product of the duration of infection and infectiousness (Fraser *et al*. 2014). We took the naïve host as the baseline case: if the RBC specialist infects a host of type *C*, we define the rate at which it transmits from that host as *B* and the duration of infection as *D*. We allowed RBC‐specialist parasites in hosts recovering from an RBC‐generalist infection (e.g. PIA treatment group) to gain the replication benefit observed in our experimental work as well as suffer the reduced survival we observed, whereas RBC‐specialist parasites in hosts with an active RBC‐generalist infection (e.g. MI group) suffer reduced survival with no replication benefit. We assume that the increase in replication translates to an increase in transmission: whereas malaria parasites produce specialised stages for transmission and may alter the rate at which they do this over the course of an infection (Reece *et al*. 2009, 2010; Pollitt *et al*. 2011; Carter *et al*. 2013), all else being equal, an increase in replication will result in the production of more transmission stages. Thus, we denote the benefit of facilitation, i.e. the proportional increase in transmission, as *f*. Finally, we approximate the decreased survival as a decrease in the duration of infection and define α as the proportional reduction in the duration of infection. The fitness of the RBC specialist in the different host classes is therefore,

$$w_{C}=BD,$$

$$w_{\mathrm{MI}}=\left(1-\alpha \right)BD,$$

$$w_{\mathrm{PIA}}=\left(1-\alpha \right)\left(1+f\right)BD.$$

Substituting these expressions into eqn (1), we found that the average fitness of an RBC specialist in a host population where the RBC generalist is circulating is thus

(2) $$\overset{¯}{w}=BD(1-\phi \alpha +f(1-\alpha )(\phi -\psi )).$$

By dividing this expression by *BD* (i.e. the fitness of the RBC specialist in the absence of the RBC‐generalist), we obtain an expression for the relative fitness of the RBC‐specialist parasite in the presence vs. absence of the RBC generalist,

(3) $${\overset{¯}{w}}_{\mathrm{rel}}=1-\phi \alpha +f\left(1-\alpha \right)\left(\phi -\psi \right).$$

**Figure 5:**
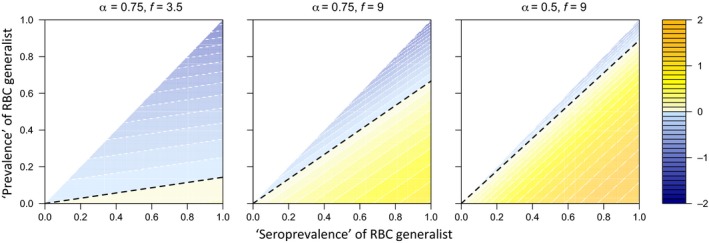
A RBC specialist can benefit from facilitation up to a threshold prevalence of a RBC generalist. Plots indicate the relative fitness of an RBC‐specialist parasite (e.g. *P. yoelii*) in the presence vs. the absence of an RBC generalist (e.g. *P. chabaudi*) over a range of costs, α, and benefits, *f*, of facilitation. Plotted values are the logged solutions to eqn (3). Within the yellow parameter space the specialist benefits from the generalist. In the blue parameter space the specialist does worse than it would alone. Note that the fitness of the generalist always decreases as the prevalence of the specialist increases. The white region reflects the fact that prevalence must be less than or equal to seroprevalence; parameter combinations above the one‐to‐one line are not plausible. The dashed line highlights the maximum prevalence (for a given seroprevalence) that obtains a benefit for *P. yoelli* fitness, given by inequality (5).

In Fig. 5, we explore how relative fitness changes with the prevalence and seroprevalence of the RBC generalist, across a range of costs and benefits to the RBC specialist. This figure demonstrates that the RBC specialist only benefits when the prevalence of the RBC generalist is below a threshold. Specifically, if

(4) 1−ϕα+f(1−α)(ϕ−ψ)>1,

then the RBC specialist benefits from the RBC generalist circulating in the host population. By rearranging this inequality, we find the equation of the dashed lines in Figure 5,

(5) $$\psi <(1-\frac{\alpha }{f(1-\alpha )})\phi$$

which gives the maximum prevalence of active generalist infection for which the RBC specialist will have higher fitness than in the absense of the RBC generalist. With some rearrangements and substitutions, we can rewrite eqn (4) as

$$\frac{p_{\mathrm{PIA}}}{p_{\mathrm{PIA}}+p_{\mathrm{MI}}}f\left(1-\alpha \right)>\alpha$$

which has an interpretation that is familiar in evolutionary biology [Hamilton's rule (Hamilton 1964a,b)]: the benefit from the RBC generalist multiplied by the probability of receiving that benefit (facilitation) must be greater than the cost.

From Fig. 5, we also see that the fitness of the RBC specialist always decreases with increasing prevalence of the RBC generalist, which can also be shown by differentiating eqn (3) with respect to ψ (i.e. the derivative is always negative). Of course, when prevalence changes, so will seroprevalence and the consequences of this for the fitness of the RBC specialist depend critically on the costs and benefits of facilitation. For example, assuming that a unit change in prevalence leads to an identical unit change in seroprevalence, then when costs are high but benefits are low (α = 0.75, *f *=* *3.5), increasing prevalence and seroprevalence leads to substantial reductions in fitness, whereas fitness reductions are more modest for other parameter combinations. Therefore, control measures aimed specifically at an RBC‐generalist malaria parasite may temporarily increase seroprevalence while decreasing prevalence, which would generate a fitness advantage to an RBC specialist, and could therefore lead to transient increases in infections with those parasites.

In reality, what matters for RBC‐specialist fitness is not the prevalence of the generalist *per se*, but the likelihood that infections with the RBC generalist have progressed to the point of altering the within‐host environment sufficiently to facilitate the RBC specialist upon its arrival in the host. Additional data would be useful for defining the transitions between host classes in a fully dynamical expansion of the model presented here (or understanding the relationship between seroprevalence and prevalence in our heuristic model). More mechanistic models (e.g. Mcqueen & Mckenzie 2006) parameterised with such data would be valuable for revealing subtleties in the interactions between *Plasmodium* species that may explain epidemiological patterns and predict off‐target consequences of interventions aimed at one species alone.

### From mice to men

We show that, by altering the within‐host environment, an RBC‐generalist parasite can facilitate the replication of an RBC specialist and bottom‐up processes are the main drivers in this interaction. Experimental manipulations of the kind reported here are, for obvious reasons, not possible with human malaria, and in natural transmission settings it is likely that patterns will be complicated further by previous infection histories and the ability of an RBC specialist (*P. vivax*) to survive as dormant stages in the host (Mueller *et al*. 2009). However, we suggest that in the many areas where *P. vivax* is circulating within the same host population as an RBC generalist (*P. falciparum*), within‐host interactions, mediated through changes in host resources, are likely to impact upon both parasite replication and host health. In particular, in areas where *P. falciparum* transmission is low, or in areas with recent outbreaks, we predict that *P. vivax* has higher fitness than in populations where it circulates alone. Our results also suggest that co‐infections of an RBC specialist and RBC generalist are highly virulent. This may come as a surprise because *P. vivax* is assumed to be comparatively benign*,* but recent studies do show that co‐infections of *P. falciparum* and *P. vivax* can result in a higher incidence of severe malaria and severe anaemia than either species alone (Genton *et al*. 2008; Tjitra *et al*. 2008). Our model also suggests *P. vivax* faces some surprising consequences of interventions targeting *P. falciparum*. If the prevalence of *P. falciparum* is high, a reduction in its prevalence could push the system into the parameter space where *P. vivax* benefits most from facilitation, generating more secondary infections than if *P. vivax* circulated alone. Of course, the reduced mortality and morbidity associated with a reduction in *P. falciparum* infections may well outweigh the clinical costs of an increase in the number of *P. vivax* infections. Whether patterns of *P. vivax* prevalence and pathogenesis fit these expectations requires urgent study.

## Conclusions

Our results highlight the importance of resource feedbacks in the interactions between co‐infecting species and the consequences of these interactions for host health and parasite epidemiology. Furthermore, our results suggest that both parasite abundance and host survival correlate with the frequency, but not the density of the preferred age of RBCs in rodent malaria infections. A similar observation has recently been made for co‐infections of the nematode *Nippostrongylus brasiliensis* and *P. chabaudi*, in which the frequency, not density, of preferred RBCs is an important predictor of infection success (Griffiths *et al*. 2015).

## Statement of authorship

RSR and SER designed the study, RSR performed the experimental work, LCP analysed the data, NM performed the modelling and all authors contributed towards writing the manuscript.

## Data Accessibility

Data pertaining to this manuscript is deposited in figshare at DOI: https://dx.doi.org/10.6084/m9.figshare.c.3262495.v1
