# Supplementary material for: Facilitation through altered resource availability in a mixed‐species rodent malaria infection
Source: Ecol Lett. 2016 Jun 30;19(9):1041–50. doi: 10.1111/ele.12639 (PMC5025717; doi:10.1111/ele.12639)
Supplement: Supplementary file 1 [file ELE-19-1041-s001.pdf]

Supplementary Information for: *"Facilitation through altered resource availability in a mixed-species rodent malaria infection"*

Text S1: Methodological details

Quantitative PCR. DNA was extracted as described in (Bell *et al.* 2006), and species-specific PCR assays used to distinguish between *P. chabaudi* and *P. yoelii*, by targeting common gametocyte gene 1 (PCHAS_062090 for *P. chabaudi* and PY02842 for *P. yoelii* in http://plasmodb.org/; (Khan *et al.* 2005; Drew & Reece 2007). The assay for *P. chabaudi*, previously published in (Wargo *et al.* 2007), has the following design: forward primer 5'-CAC AAT ATA GTA TAA AAG TAG GAC TTG AAA ATA ATA GTA G-3', reverse 5'-GGA ATA TGG GAT ATT GTC AAA GGA TAT AC-3', probe (TaqMan MGB) 5'-6FAM–TTT TCC ACT TAC AAC TCC A-3'. The *P. yoelii* assay was custom made for this experiment by PrimerDesign, Co and has the following design: forward 5'-CTG TAA GGC CAT TTA AGG GGG-3', reverse 5'- TCA TAG TAC CAT TCT TTT TGC TTT TTC-3', probe (TAMRA) 5'-6FAM-CTA AGT CAT CAT TTA TAA CCT TTA CAG CAT CAT CAT-3'. We performed qPCR reactions as described in (Bell *et al.* 2006; Wargo *et al.* 2007) for *P. chabaudi* and following PrimerDesign's guidelines for *P. yoelii.* Briefly, for *P. yoelii*, reactions were set up at a final volume of 20 µl, using 5 µl DNA, 10 µl of 2x TaqMan universal PCR master mix (Applied Biosystems), 4 µl of water and 1 µl of the primer/probe mix provided by PrimerDesign (cycling conditions: 95 ºC for 10 min.; 50 cycles at 95 ºC for 15 s and 60 ºC for 1min.). We validated both assays before analysing the experimental samples to confirm that: (i) both assays are species-specific, with no cross-reactivity; (ii) the sensitivity of each assay is not affected by the density of heterospecifics; (iii) both assays show low interassay variation (*P. yoelii:* 0.4-2.8 %; P. *chabaudi*: 0.5-0.8 %; (Khan *et al.* 2005; Schneider *et al.* 2005; Drew & Reece 2007)); and (iv) the detection limit is low and similar at 2-5 and 3-5 parasites/µl for *P. yoelii* and *P. chabaudi,* respectively.

Immunological assays. We measured antibodies of the IgG2a class using enzyme linked immunosorbent assays (ELISA) against *P. chabaudi* and *P. yoelii*. We prepared crude antigen homogenate from *P. yoelii* parasitized erythrocytes, following an adapted protocol from (Nie *et al.* 2009). Briefly, we bled *P. yoelii* infected mice by cardiac puncture into heparinised syringes and diluted blood 1:2 in RPMI medium. This was underlayed in 67 % Percoll:RPMI and centrifuged at 1500 xg (10 min.). We then harvested late stage parasites from the interface, washed them in RPMI and centrifuged at 2000 rpm (5 min.). We then incubated parasites in pre-warmed trypsin at 37 ºC (to cleave antibodies) and washed cells twice with RPMI by centrifuging at 2000 rpm (5 min.) We then re-suspended cells in pre-warmed 0.05 % saponin to lyse RBC membranes, centrifuged this at 2000 rpm (10 min.), washed the supernatant in PBS until this became clear and lysed parasites with 5 cycles of freeze-thawing. Finally we measured protein concentration using a Bradford assay (BioRad).

To measure IgG2a against *P. yoelii*, we first coated plates (Immunosorb, NUNC) with 50 µl (per well) of 1 µg/ml of crude antigen homogenate in carbonate buffer and incubated them overnight at 4 ºC. We then blocked non-specific binding with 100 µl of 5% Marvel:carbonate buffer and incubated this at 37 ºC for 2h. We washed wells 5 times with Tris buffered saline with 0.1 % Tween (TBST) after each incubation. We then added serum samples at dilutions from 1:100 to 1:800 and incubated plates for 2h at 37 ºC. After this, we filled wells with 100 µl of 1µg/ml Biotin Rat Anti-Mouse IgG2a detection antibody (BD Biosciences) diluted in TBST:1%Bovine serum albumine (BSA) and incubated plates for 1h at 37 ºC. Finally, we diluted extravidin peroxidase 1/8000 in TBST:1 %BSA (100 µl per well), incubated plates for 30 min. at 37 ºC and developed samples with 100 µl per well of TMB for 20 min. We stopped reactions with 100 µl of 1M HCL and read plates at 405 nm. All data are presented from a dilution at which all samples are in the linear range of the curve. For the *P. chabaudi* assay we followed the same protocol, with some exceptions: plates were coated with *P. chabaudi* MSP1, non-specific binding was blocked by 5 % BSA:carbonate buffer, horseradish peroxidase conjugated goat anti-mouse IgG2a (Southern Biotech) was used as the detection antibody and no extravidin peroxidase step was performed.

Supplementary Figures and Tables

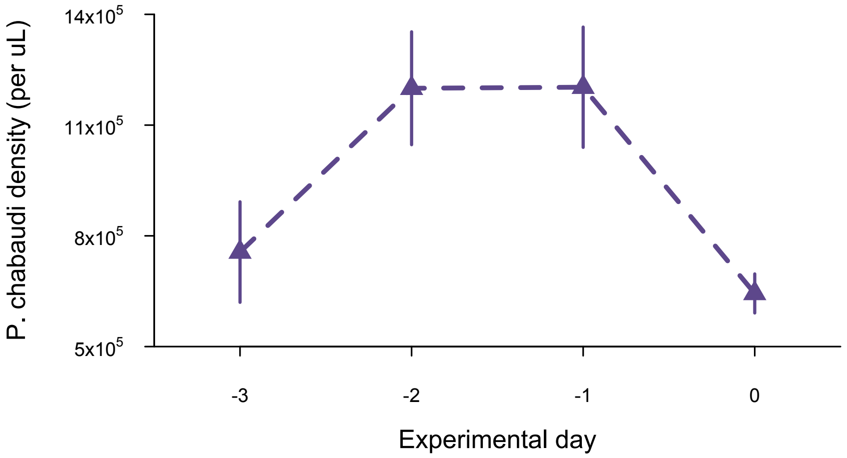

Figure S1: Density of *P. chabaudi* on the four days prior to *P. yoelii* infection in the parasite induced anaemia treatment group. *P. yoelii* infections were initiated after the measurement on day 0. Error bars show the standard error of the mean.

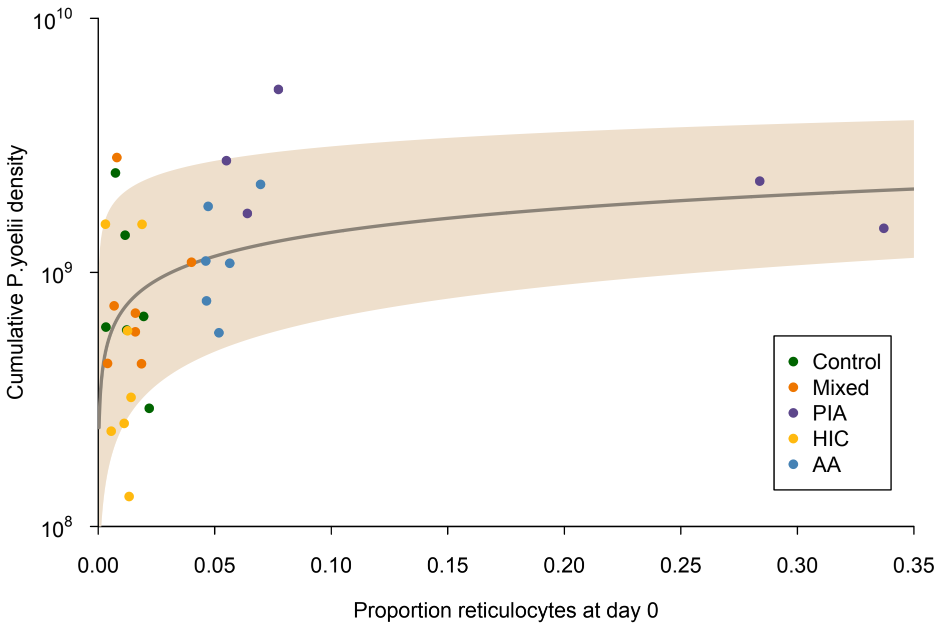

Figure S2: *P. yoelii* density positively correlates with proportion of reticulocytes. Grey line shows the line of best fit from a generalised linear model and the shaded area indicates the standard error of the model estimate. Relationship is linear on a log-log scale.

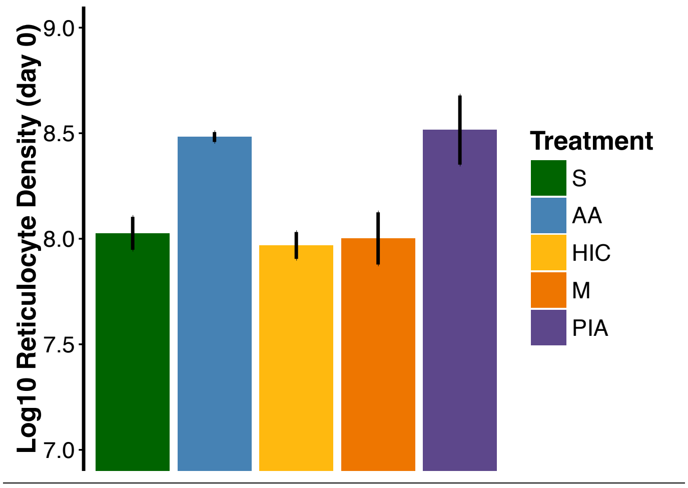

Figure S3: Reticulocyte density on day 0 (the day of *P. yoelii* infection). Mean ± standard error.

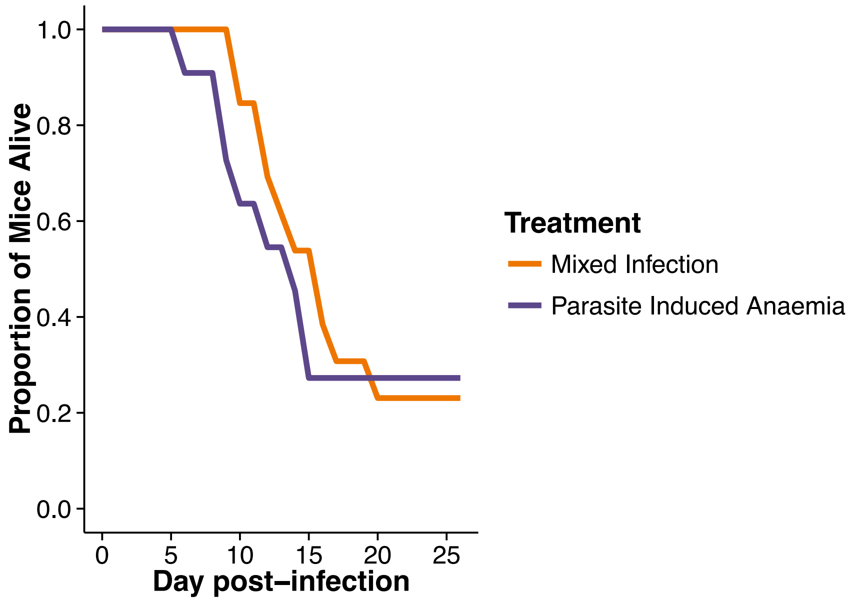

Figure S4: Proportion of mice alive for 26 days after *P. yoelii* infection. Treatments represented are the high mortality treatments, i.e. the infections containing both *P. chabaudi* and *P. yoelii*.

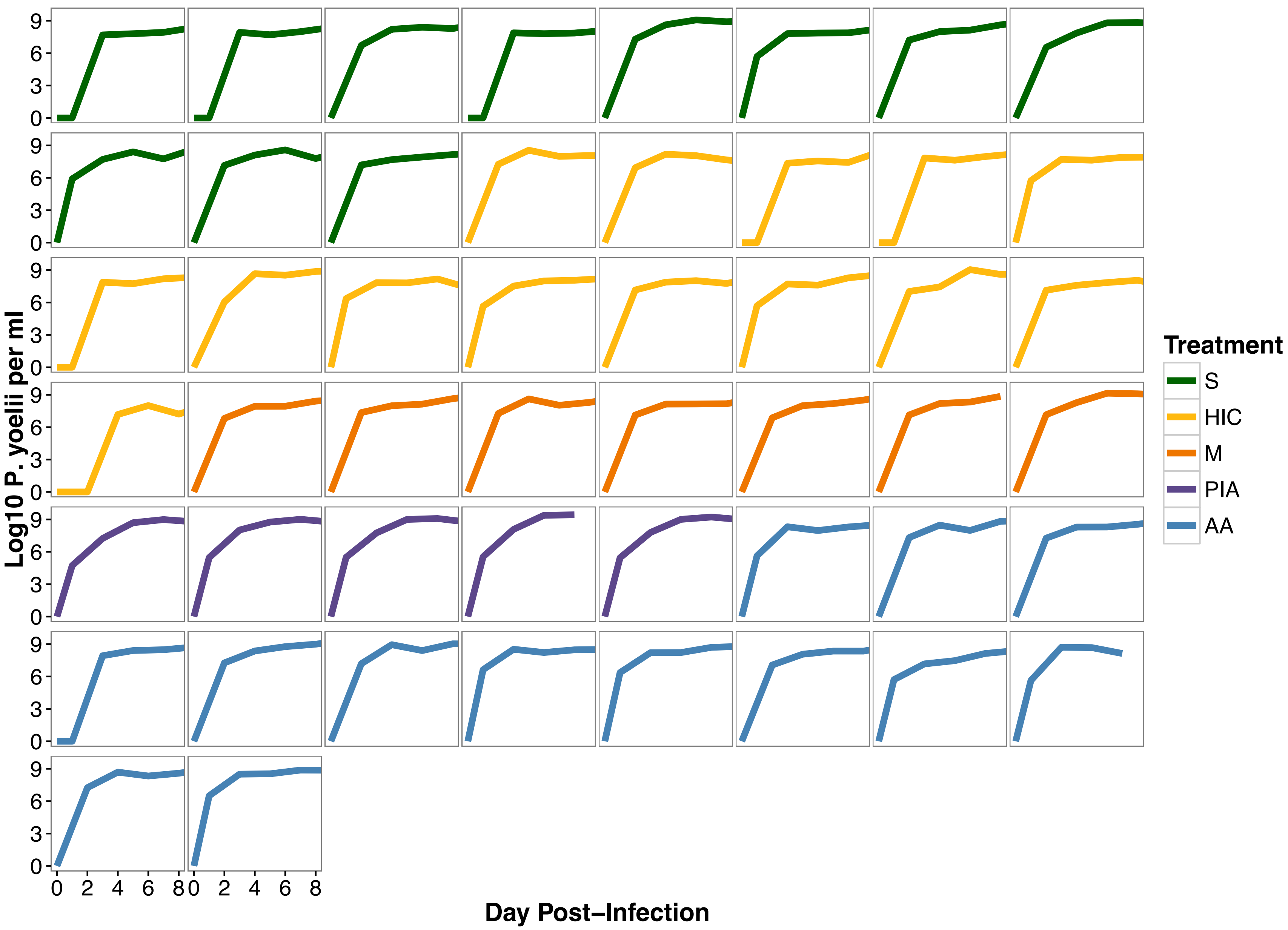

Figure S5: *P. yoelii* infection dynamics. Each panel represents the parasite counts for a different mouse.

Table S1: Treatment differences in the within-host environment on day 0.

**log10 red blood cells ~ treatment**		**d.f. = 4, 45**	**F = 188.77**	**p < 0.0001**
Pairwise comparisons			z	p
Control (single infection)	vs.	Mixed infection	0.507	0.986
		Parasite induced anaemia	**22.789**	**< 0.0001**
		Heterologous immune challenge	2.031	0.246
		Artificial anaemia	**14.249**	**< 0.0001**
Mixed infection	vs.	Parasite induced anaemia	**20.573**	**< 0.0001**
		Heterologous immune challenge	1.238	0.724
		Artificial anaemia	**11.929**	**<0.0001**
Parasite induced anaemia	vs.	Heterologous immune challenge	**22.022**	**< 0.0001**
		Artificial anaemia	**12.265**	**< 0.0001**
Heterologous immune challenge	vs.	Artificial anaemia	**13.031**	**< 0.0001**
**log10 reticulocytes ~ treatment**		**d.f = 4, 45**	**F = 12.02**	**p < 0.0001**
Pairwise comparisons			z	p
Control (single infection)	vs.	Mixed infection	0.206	0.9995
		Parasite induced anaemia	**3.721**	**0.002**
		Heterologous immune challenge	0.586	0.9766
		Artificial anaemia	**4.569**	**< 0.0001**
Mixed infection	vs.	Parasite induced anaemia	**3.598**	**0.0028**
		Heterologous immune challenge	0.294	0.9983
		Artificial anaemia	**4.205**	**0.0002**
Parasite induced anaemia	vs.	Heterologous immune challenge	**4.305**	**0.0002**
		Artificial anaemia	0.257	0.9990
Heterologous immune challenge	vs.	Artificial anaemia	**5.473**	**< 0.0001**
**logit proportion retics ~ treatment**		**d.f = 4, 45**	**F = 30.79**	**p < 0.0001**
Pairwise comparisons			z	p
Control (single infection)	vs.	Mixed infection	0.112	1
		Parasite induced anaemia	**7.586**	**<0.001**
		Heterologous immune challenge	0.243	0.9992
		Artificial anaemia	**6.729**	**<0.001**
Mixed infection	vs.	Parasite induced anaemia	**7.08**	**<0.001**
		Heterologous immune challenge	0.095	1
		Artificial anaemia	**5.995**	**<0.001**
Parasite induced anaemia	vs.	Heterologous immune challenge	**8.042**	**<0.001**
		Artificial anaemia	2.537	0.0805
Heterologous immune challenge	vs.	Artificial anaemia	**7.411**	**<0.001**
log10 *P. yoelii* antibodies ~ treat		d.f = 4, 45	F = 2.15	p = 0.09
**log10 *P. chabaudi* antibodies ~ treat**		**d.f = 4, 42**	**F = 5.82**	**p < 0.001**
Pairwise comparisons			z	p
Control (single infection)	vs.	Mixed infection	0.443	0.9918
		Parasite induced anaemia	**3.08**	**0.0173**
		Heterologous immune challenge	**3.936**	**<0.001**
		Artificial anaemia	0.925	0.8855
Mixed infection	vs.	Parasite induced anaemia	2.471	0.0952
		Heterologous immune challenge	**2.963**	**0.0247**
		Artificial anaemia	0.385	0.9953
Parasite induced anaemia	vs.	Heterologous immune challenge	0.145	0.9999
		Artificial anaemia	2.296	0.1434
Heterologous immune challenge	vs.	Artificial anaemia	**2.854**	**0.0341**

Table S2: Treatment effects on the cumulative density of *P. yoelii*

**Single and Mixed infections (Figure 2; PCR data)**				
**log10 *P. yoelii* ~ treatment**		**d.f. = 2, 15**	**F = 5.46**	**p = 0.017**
Tukey post-hoc pairwise comparisons			z	p
Control (single infection)	vs.	Mixed infection	0.034	0.9994
		Parasite induced anaemia	**2.854**	**0.0119**
Mixed infection	vs.	Parasite induced anaemia	**2.984**	**0.0079**
**Single *P. yoelii* infections in mice with different starting within-host environments (Figure 3; PCR and slide data)**				
**log10 *P. yoelii* ~ treatment*method (PCR or microscopy)**		**d.f. = 2, 32**	**F = 0.8014**	**p = 0.457**
**log10 *P. yoelii* ~ method (PCR or microscopy)**		**d.f. = 1, 34**	**F = 14.571**	**p < 0.001**
**log10 *P. yoelii* ~ treatment**		**d.f. = 2, 34**	**F = 9.1347**	**p < 0.001**
Tukey post-hoc pairwise comparisons			z	p
Control (single infection)		Heterologous immune challenge	1.393	0.344
		Artificial anaemia	**2.602**	**0.025**
Heterologous immune challenge	vs.	Artificial anaemia	**4.230**	**< 0.001**

Table S3: Within-host environmental effects on *P. yoelii* density. Densities measured by PCR.

log10 *P.y* density (week P.I) ~			
Term	d.f. for term, residual d.f.	F	p
Reticulocyte density on day 0	1, 26	0.05	0.82
P. yoelii antibodies	1, 27	0.17	0.70
P. chabaudi antibodies	1, 28	0.22	0.65
**Proportion of reticulocytes on day 0**	**1, 29**	**6.4**	**0.017**

Table S4: Treatment effects on host mortality

**cbind (dead,n) ~ treatment**				
**Collapsing groups to test for pairwise comparisons**	**d.f. , n**	χ^2	p	**AIC**
5 Treatment levels: Control, AA, PIA, Mixed, HIC	4, 63	23.11	< 0.0005	18.95
4 Treatment levels: (Control + HIC), AA, PIA, Mixed	3, 63	23.11	< 0.0005	16.95
3 Treatment levels: (Control + HIC + AA), PIA, Mixed	2, 63	21.01	< 0.0005	15.00
**2 Treatment levels: (Control + HIC + AA), (PIA + Mixed)**	**1, 63**	**21.01**	**< 0.0005**	**10.17**

Table S5: Treatment effects on host anaemia and weight loss.

Minimum weight ~ treat		d.f = 4, 63	F = 4.15	p = 0.0051
Pairwise comparisons			z	p
Control (single infection)	vs.	Mixed infection	1.31	0.68
		Parasite induced anaemia	2.05	0.24
		Heterologous immune challenge	1.44	0.60
		Artificial anaemia	1.23	0.073
Mixed infection	vs.	Parasite induced anaemia	0.86	0.91
		Heterologous immune challenge	**2.94**	**0.028**
		Artificial anaemia	0.06	1
Parasite induced anaemia	vs.	Heterologous immune challenge	**3.62**	**<0.005**
		Artificial anaemia	0.91	0.89
Heterologous immune challenge	vs.	Artificial anaemia	1.23	0.73
Minimum RBC density ~ treat		d.f = 4, 63	F = 6.20	p < 0.0005
Pairwise comparisons			z	p
Control (single infection)	vs.	Mixed infection	1.69	0.44
		Parasite induced anaemia	1.99	0.27
		Heterologous immune challenge	1.76	0.39
		Artificial anaemia	2.12	0.21
Mixed infection	vs.	Parasite induced anaemia	0.42	0.99
		Heterologous immune challenge	**3.68**	**<0.005**
		Artificial anaemia	0.49	0.98
Parasite induced anaemia	vs.	Heterologous immune challenge	**3.87**	**<0.005**
		Artificial anaemia	0.05	1
Heterologous immune challenge	vs.	Artificial anaemia	**4.10**	**<0.001**

Table S6: Predictors of death in high virulence treatments (PIA and MI)

Mortality ~			
	d.f.	χ^2	p
Minimum weight	1, 20	0.18	0.67
Minimum red blood cell count	1, 21	3.49	0.062
**Mean proportion reticulocytes**	**1 , 22**	**15.20**	**<0.0001**
**Treatment**	**1 , 22**	**8.38**	**<0.005**

References

1.Bell, A.S., De Roode, J.C., Sim, D. & Read, A.F. (2006). Within-host competition in genetically diverse malaria infections: parasite virulence and competitive success. *Evolution*, 60, 1358–1371.
2.Drew, D.R. & Reece, S.E. (2007). Development of reverse-transcription PCR techniques to analyse the density and sex ratio of gametocytes in genetically diverse Plasmodium chabaudi infections. *Molecular and Biochemical Parasitology*, 156, 199–209.
3.Khan, S.M., Franke-Fayard, B., Mair, G.R., Lasonder, E., Janse, C.J., Mann, M., *et al.* (2005). Proteome analysis of separated male and female gametocytes reveals novel sex-specific Plasmodium biology. *Cell*, 121, 675–687.
4.Nie, C.Q., Bernard, N.J., Norman, M.U., Amante, F.H., Lundie, R.J., Crabb, B.S., *et al.* (2009). IP-10-Mediated T Cell Homing Promotes Cerebral Inflammation over Splenic Immunity to Malaria Infection. *Plos Pathogens*, 5, e1000369.
5.Schneider, P., Wolters, L., Schoone, G., Schallig, H., Sillekens, P., Hermsen, R., *et al.* (2005). Real-time nucleic acid sequence-based amplification is more convenient than real-time PCR for quantification of Plasmodium falciparum. *JOURNAL OF CLINICAL MICROBIOLOGY*, 43, 402–405.
6.Wargo, A.R., De Roode, J.C., Huijben, S., Drew, D.R. & Read, A.F. (2007). Transmission stage investment of malaria parasites in response to in-host competition. *Proc. Biol. Sci.*, 274, 2629–2638.
